# Reduction of motorcycle-related deaths over 15 years in a developing country

**DOI:** 10.1186/s13017-022-00426-y

**Published:** 2022-04-29

**Authors:** Yasin J. Yasin, Hani O. Eid, David O. Alao, Michal Grivna, Fikri M. Abu-Zidan

**Affiliations:** 1 Institute of Public Health, College of Medicine and Health Sciences, United Arab Emirates University, Al-Ain, United Arab Emirates; 2 Department of Environmental Health and Behavioral Sciences, School of Public Health, College of Health Sciences, Mekelle University, Mekelle, Ethiopia; 3 Rescue and Air Ambulance, Abu Dhabi Police Aviation, Abu Dhabi, United Arab Emirates; 4 Department of Internal Medicine, College of Medicine and Health Sciences, United Arab Emirates University, Al-Ain, United Arab Emirates; 5 Emergency Department, Al-Ain Hospital, Al-Ain, United Arab Emirates; 6 Department of Public Health and Preventive Medicine, Second Faculty of Medicine, Charles University, Prague, Czech Republic; 7 Department of Surgery, College of Medicine and Health Sciences, United Arab Emirates University, Al-Ain, United Arab Emirates

**Keywords:** Road traffic collision, Motorcycle, Trauma, Injury, Death, Incidence, United Arab Emirates

## Abstract

**Background:**

There have been major improvements in the trauma system and injury prevention in Al-Ain City. We aimed to study the impact of these changes on the incidence, pattern, injury severity, and outcome of hospitalized motorcycle-related injured patients in Al-Ain City, United Arab Emirates.

**Methods:**

This is a retrospective analysis of two separate periods of prospectively collected data which were retrieved from Al-Ain Hospital Trauma Registry (March 2003 to March 2006 compared with January 2014 to December 2017). All motorcycle-injured patients who were admitted to Al-Ain Hospital for more than 24 h or died in the Emergency Department or after hospitalization were studied.

**Results:**

The incidence of motorcycle injuries dropped by 37.1% over the studied period. The location of injury was significantly different between the two periods (*p* = 0.02, Fisher's exact test), with fewer injuries occurring at streets/highways in the second period (69.1% compared with 85.3%). The anatomical injury severity of the head significantly increased over time (*p* = 0.03), while GCS on arrival significantly improved (*p* < 0.0001), indicating improvements in both prehospital and in-hospital trauma care. The mortality of the patients significantly decreased (0% compared with 6%, *p* = 0.002, Fisher's exact test).

**Conclusions:**

The incidence of motorcycle injuries in our city dropped by almost 40% over the last 15 years. There was a significant reduction in the mortality of hospitalized motorcycle-injured patients despite increased anatomical severity of the head injuries. This is attributed to improvements in the trauma care system, including injury prevention, and both prehospital and in-hospital trauma care.

## Introduction

Road traffic collisions (RTCs) are a major persistent public health problem. Out of 1.35 million deaths worldwide, 28% were caused by motorized 2–3 wheelers [1]. Motorized 2–3 wheelers account for 1.6% of all registered vehicles in the United Arab Emirates (UAE) [1]. During 2016–2017, they caused 6% of road traffic deaths in the UAE and 2% in the Emirate of Abu Dhabi [1, 2]. Motorcycle riders are thirty times more likely to die in RTCs compared with other road users because of the high energy transfer to their exposed bodies when colliding with high speed [3, 4]. This causes severe head injuries and increases death [4–8]. Motorcycle-related injuries had the highest mortality of hospitalized trauma patients in our city, of whom more than 40% had head injuries [6].

Al-Ain City is growing rapidly (65% growth over the last 15 years). It currently has a population of around three quarters of a million [9–11]. Its main public hospital (Al-Ain Hospital) had treated around 80% of the trauma patients of our city before the COVID-19 pandemic (March 2020), after which it was allocated to treat only COVID-19 patients [12, 13]. Improving trauma systems reduces trauma mortality [14–16]. A trauma system is defined as a “preplanned, organized and coordinated injury-control effort in a defined geographic area” [17]. In 2001, a Trauma Group was established in Al-Ain City with a clear preplanned, organized and coordinated effort to improve trauma outcomes. Since then, there have been major developments in the trauma system in our city including injury prevention, prehospital care, in-hospital trauma management, trauma registries, trauma research and trauma education. This included establishing Al-Ain Hospital Trauma Registry [18, 19] which generated significant amount of useful data on trauma management and epidemiology that resulted in numerous high-quality scientific publications. These publications were used, through the media, to promote and improve road, work-related, and home safety [20, 21] as a step toward establishing a trauma system [22]. Injury prevention interventions included installation of speed cameras, high penalties for violations, and enforcing labor safety [23–25]. Educational activities included establishing the ATLS [26] and Point-of-Care Ultrasound (POCUS) courses [27] which became an integral part of our clinical practice [28]. Improvements occurred in the prehospital transport system, and in-hospital trauma management with increased number of EMS trained staff [2], mandatory ATLS training for trauma teams [26], having 24-h angioembolization and interventional radiology, following trauma management updates including hypotensive resuscitation, using POCUS, and damage control surgery [28–30].

We have previously shown that the maturity of our trauma system reduced trauma death [16], but we did not study its effects on motorcycle-related injuries. Hereby we aim to study the impact of the trauma system development on the incidence, injury pattern and severity, and outcome of hospitalized motorcycle-injured patients in Al-Ain City, United Arab Emirates.

## Patients and methods

### Ethical considerations

Ethical approval was obtained from the Human Research Ethics Committee of Al-Ain Hospital, Al-Ain, United Arab Emirates (AAHEC-03-20-008). Written informed consent was obtained from the patients or their caregivers to use the data for this study.

### Study design

This is a retrospective analysis of prospectively collected data which were retrieved from Al-Ain Hospital Trauma Registry. Data were collected, coded, and entered by full-time trained research fellows and nurses.

### Patients

We studied all motorcycle-related injured patients who were admitted for more than 24 h or who died on arrival at the Emergency Department or after hospitalization from March 2003 to March 2006 (first period, 3 years) and January 2014 to December 2017 (second period, 4 years).

### Studied variables

Studied variables included demography, location of injury, mode of arrival, vital signs and Glasgow Coma Scale (GCS) on arrival, severity of the injury of regions by Abbreviated Injury Scale (AIS), Injury Severity Score (ISS), New Injury Severity Score (NISS), length of ICU stay, length of ventilation days, length of hospital stay, and clinical outcome.

### Setting

Al-Ain City is the second largest city in the Abu Dhabi Emirate, UAE. The city had about 463,000 residents during the first period [10, 11] and around 767,000 residents during the second period [9]. Al-Ain Hospital is one of the two major public hospitals in Al-Ain City, which treated around 80% of the hospitalized trauma patients during the study periods [12, 13]. It provided a wide range of general and specialized clinical services with 412 beds [31].

### Calculations

Since Al-Ain Hospital treated 80% of the RTC injured patients in Al-Ain City during the studied periods [12], the correction factor for this percentage would be 100 divided by 80, which equals 1.25. Accordingly, the standardized incidence rate was calculated as follows: (1.25 × annual patients)/(population/100,000).

### Statistical analysis

Data are presented as numbers (percentage) for categorical data or median (range) for continuous or ordinal data. Fisher's exact test was used to compare categorical data for two independent groups, while Mann–Whitney *U* test was used to compare continuous or ordinal data of two independent groups. Data were analyzed with the IBM SPSS Statistics version 26 (SPSS Inc, Chicago, IL, USA). A *p* value of less than 0.05 was accepted as statistically significant.

## Results

There were 68 patients during the first period and 94 patients during the second period. This gives an annual incidence of 6.2/100,000 population for the first period compared with 3.9/100,000 population for the second period, a reduction of 37.1%. Table 1 shows the demography of the two periods. There was no significant difference in age, gender, nationality, or mode of arrival. Nevertheless, the location of injury was significantly different between the two periods (*p* = 0.02, Fisher's exact test). There were relatively fewer street/highway injuries during the second period (69.1% compared with 85.3%), while other areas of less speed limits increased like homes (7.4% compared with 0%), workplace (3.2% compared with 0%), and public areas (4.3% compared with 0%).

**Table 1:** Demographic characteristics and injury location of hospitalized motorcycle-injured patients during the period 2003–2006 (*n* = 68) and 2014–2017 (*n* = 94), Al-Ain Hospital, Al-Ain United Arab Emirates

Variable	Years 2003–2006 (*n* = 68)	Years 2014–2017 (*n* = 94)	*p* value
Age (years)	27 (4–64)	27.5 (3–86)	0.49
Gender			0.99
Male	66 (97.06%)	90 (95.74%)	
Nationality			0.87
UAE nationals	26 (38.24%)	34 (36.17%)	
Non-UAE	41 (60.29%)	58 (61.7%)	
Location of injury			0.02
Home	0 (0%)	7 (7.4%)	
Street/highway	58 (85.3%)	65 (69.1%)	
Workplace	0 (0%)	3 (3.2%)	
Off-roads	10 (14.7%)	14 (14.9%)	
Public area	0 (0%)	4 (4.3%)	
Other	0 (0%)	1 (1.1%)	
Mode of arrival			0.16
Ambulance	42 (61.76%)	61 (64.89%)	
Private car	26 (38.24%)	22 (23.40%)	

Data are presented as number (percentage) or median (range), *p* value = Fisher's Exact test, or Mann–Whitney *U* test as appropriate

**Fig. 1:**
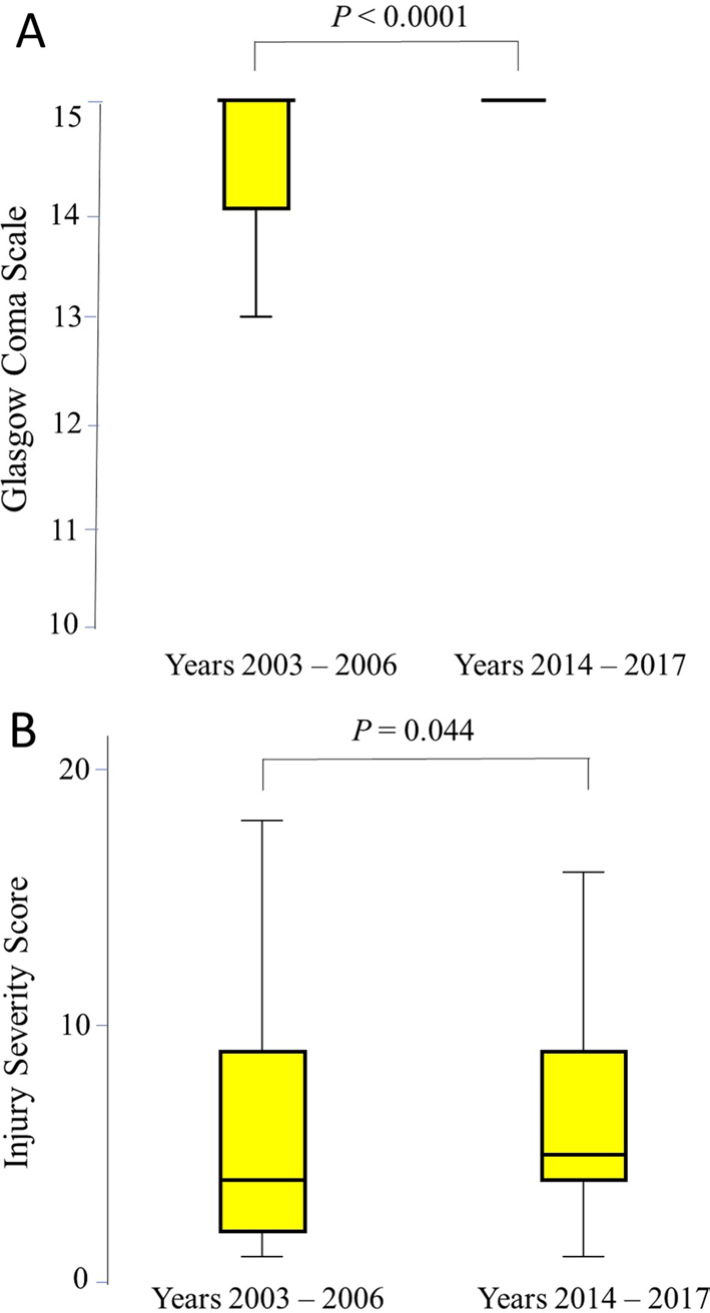
Box-and-whisker plot of Glasgow Coma Scale and Injury Severity Score of hospitalized motorcycle-injured patients during the period 2003–2006 (*n* = 68) and 2014–2017 (*n* = 94), Al-Ain Hospital, Al-Ain, United Arab Emirates. The box represents the 25th to the 75th percentile IQR. The horizontal line within the box represents the median. *p* value = Mann–Whitney *U* test

Table 2 shows the severity markers of injuries. GCS was significantly higher in the second period compared with the first period ((median (range), mean (SD): 15 (5–15), 14.69 (1.52) compared with 15 (3–15), 13.29 (3.69) *p* < 0.0001) (Fig. 1) despite having significantly higher ISS (median (range): 5 (1–41) compared with 4 (1–29), *p* = 0.04), and NISS (median (range): 9 (1–41) compared with 5.5 (1–41), *p* = 0.01. The length of ICU and length of ventilation days significantly decreased in the second period (median (range): 0 (0–32) days compared with 4 (1–19) days, *p* < 0.0001; 0 (0–32) days compared with 1.5 (0–12) days, *p* < 0.0001, respectively) (Table 2). Nevertheless, there was no significant difference in the length of hospital stay between the two periods (*p* = 0.12).

**Table 2:** Injury severity markers of motorcycle-injured hospitalized patients during the period 2003–2006 (*n* = 68) and 2014–2017 (*n* = 94), Al-Ain Hospital, Al-Ain, United Arab Emirates

Variable	Years 2003–2006 (*n* = 68)	Years 2014–2017 (*n* = 94)	*p* value
SBP mmHg	138 (96–180)	135 (94–197)	0.54
Heart rate (bpm)	90 (60–155)	88 (62–168)	0.79
RR per minute	22 (15–27)	18 (12–28)	0.11
GCS*	15 (3–15)	15 (5–15)	< 0.0001
	13.29 (3.69)	14.69 (1.52)	
ISS	4 (1–29)	5 (1–41)	0.04
NISS	5.5 (1–41)	9 (1–41)	0.01
ICU stay (days)	4 (1–19)	0 (0–32)	< 0.0001
Ventilation (days)	1.5 (0–12)	0 (0–32)	< 0.0001
Total hospital stay (days)	5 (1–79)	4 (2–43)	0.12
Death	4 (5.9%)	0 (0%)	0.002

Data are presented as number (percentage) or median (range), *p* value = Fisher's Exact test, or Mann–Whitney *U* test as appropriate

*SBP* systolic blood pressure, *RR* respiratory rate, *GCS* Glasgow Coma Scale, *ICU* intensive care unit, *ISS* Injury Severity Score, *NISS* New Injury Severity Score, *bpm* beats per minute

*GCS presented both as median (range) and mean (SD)

Table 3 shows the anatomical injured regions and their AIS of the two periods. The second period had a significantly lower percentage of head injuries (19.2% compared with 35.3%, *p* = 0.03, Fisher's exact test) and a significantly higher percentage of neck injuries (9.6% compared with 0%, *p* = 0.01, Fisher's exact test). The AIS was significantly higher in both the head and upper extremities during the second period (median (range: 3 (1–5) compared with 1.5 (1–4), *p* = 0.03), and median (range): 2 (1–3) compared with 1 (1–2), *p* < 0.0001, respectively). The mortality significantly decreased during the second period (0% compared with 5.9%, *p* = 0.002, Fisher's exact test) (Table 2).

**Table 3:** Comparison of injured anatomical regions of motorcycle-related injured hospitalized patients during the period 2003–2006 (*n* = 68) and 2014–2017 (*n* = 94), Al-Ain Hospital, Al-Ain, United Arab Emirates

Region	Anatomical region			Abbreviated Injury Scale (AIS)		
	Years 2003–2006 (*n* = 68)	Years 2014–2017 (*n* = 94)	*p* value	Years 2003–2006 (*n* = 68)	Years 2014–2017 (*n* = 94)	*p* value
Head	24 (35.3%)	18 (19.2%)	0.03	1.5 (1–4)	3 (1–5)	0.03
Face	20 (29.4%)	19 (20.2%)	0.20	1 (1–2)	1 (1–2)	0.13
Neck	0 (0%)	9 (9.6%)	0.01	–	1 (1–1)	–
Chest	14 (20.6%)	22 (23.4%)	0.71	2 (1–3)	2 (1–4)	0.95
Abdomen	3 (4.4%)	12 (12.8%)	0.10	1 (1–2)	2 (1–3)	0.63
Spine	5 (7.4%)	10 (10.6%)	0.59	2 (2–2)	2 (1–2)	0.99
Upper extremities	35 (51.5%)	41 (43.6%)	0.34	1 (1–2)	2 (1–3)	0.0001
Lower extremities	32 (47.1%)	45 (47.9%)	0.99	2 (1–3)	2 (1–3)	0.51

Data are presented as numbers (%)

*p* value = Fisher's Exact test

## Discussion

Our study has shown a significant improvement in the outcome of hospitalized motorcycle-related injured patients over the last 15 years. The anatomical injury severity of the head doubled while GCS on arrival improved. This indicates better prehospital and in-hospital trauma care. The mortality dropped from 6% to none. Furthermore, the incidence of motorcycle-related injuries dropped by almost 40% in our city indicating improvements in the injury prevention in our setting. These findings highlight the impact of the development of our trauma system in reducing the injury incidence and improving the clinical outcomes of motorcycle-related injured patients.

Globally, motorcycle-related deaths are quarter of all RTC deaths [1, 32–35]. It is expected to increase by 11% worldwide over the coming 10 years [36]. The United Nations' global aim was to reduce road deaths by half over 2010 to 2020 [37]. Interestingly, this was achieved in our setting [14, 38, 39] but not globally [40]. The effect size and time of improvement vary between different countries [38, 39]. The effect size in our study is large compared with a multicenter study from Israel which showed reduced mortality by 43% [14]. However, our study stemmed from a single hospital. These results can be attributed to improvements in the EMS prehospital care in the Abu Dhabi Emirate [14, 41], which was evidenced by the improved GCS of injured patients on arrival. The reduced mortality despite the increased anatomical severity of the head injury reflects the improved trauma care within our hospital over the last 15 years. These developments include establishing a trauma team that attend with every major trauma, following a trauma management protocol, a 24 h availability of a 16-slice CT scanner and radiologist adjacent to the Emergency Department, development of a trauma CT protocol, availability of 24/7 angioembolization suite run by expert interventional radiologists, presence of an on-call neurosurgical team, establishing an expanded state-of-the-art intensive care unit that follow well-developed guidelines, collecting data on trauma management, continuous clinical audit, and following a quality improvement program.

**Fig. 2:**
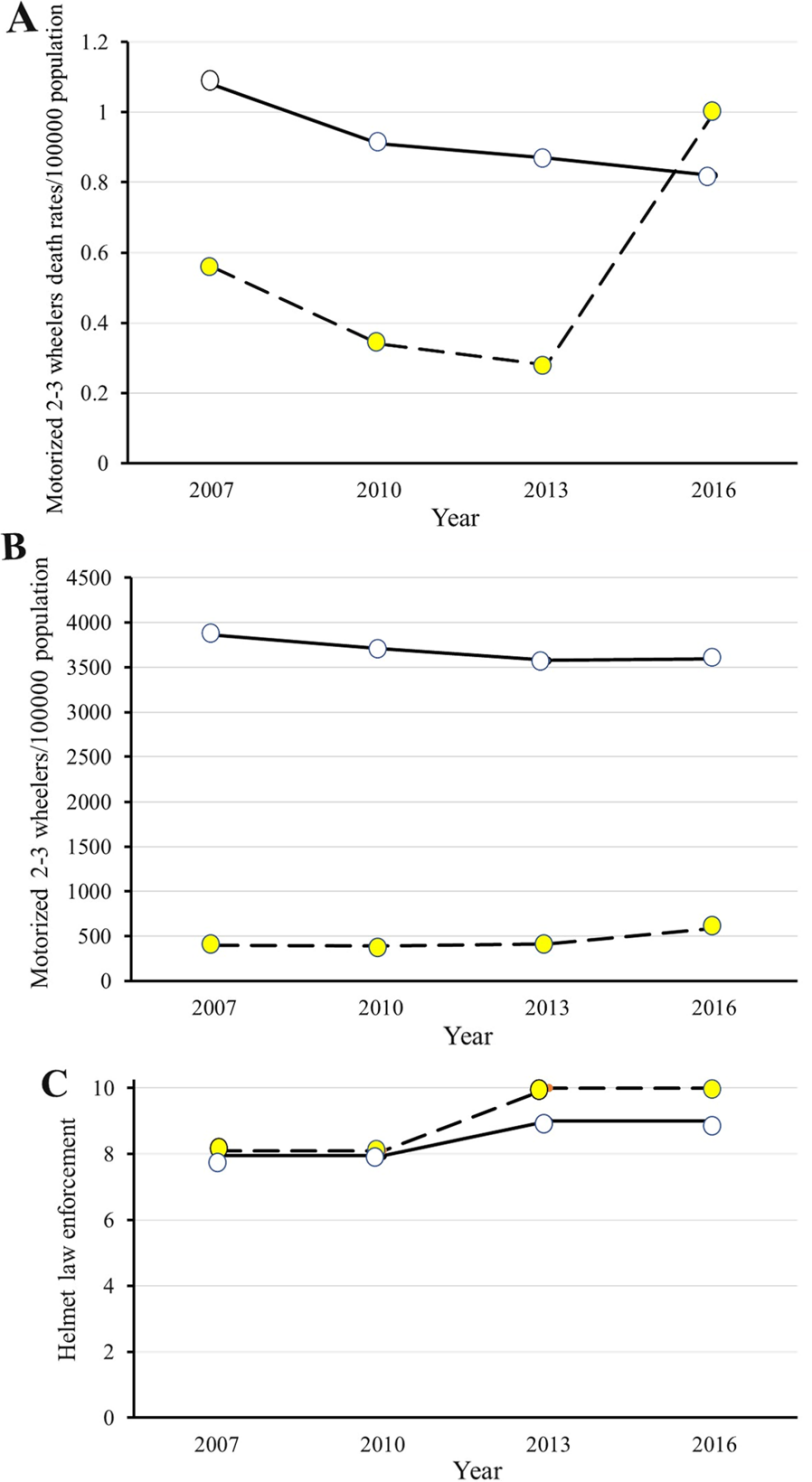
Comparison of motorized 2–3 wheelers death rate/100,000 population (**A**), motorized 2–3 wheelers/100,000 population (**B**), and helmet law enforcement (on a scale of 0 to 10) (**C**) 2007–2016 between UAE (yellow circles) and high-income countries (white circles). Data were collected and analyzed by the authors. *Source*: WHO Global status report on road safety 2007–2016 published over 2009–2018 [1, 32–34]

Over the last two decades, there have been tremendous improvements in injury prevention measures in the UAE. These included enforcement of safety regulations (such as helmet and speed law enforcement), use of safety devices (like helmet usage), installation of road speed cameras, penalties on speeding violations, and educational programs [1, 23, 24, 32–34]. This explains the reduction of the percentage of head injuries in the second period by 25% in our study. Although our city previously used motorcycles less than four-wheel vehicles [6, 42], we have observed their recent increase as a cheap transportation and food delivery tool. Figure 2 compares motorized 2–3 wheelers mortality rate, standardized number of motorized 2–3 wheelers, and helmet law enforcement between UAE and other high-income countries [1, 32–34]. The motorcycle-related death rate increased sharply in the UAE from 2013 to 2016, which cannot be explained by the minimum increase in the number of motorcycles used in the UAE (Fig. 2). Modernization, improvement, and maturity of our trauma system in all its components contributed to the improved clinical outcome in the current study [16]. The increased severity of head injuries in our study may indicate low helmet compliance, low-quality helmets, or improperly fastened helmets [7, 43, 44]. Collisions became less in high-speed streets/highways and increased in low-speed residential areas, which may explain this finding because riders may be less careful in using their helmets in these areas. Developing an injury prevention strategy to address the concerns regarding the quality of the helmets and collisions in the residential areas is important.

It may be surprising to notice that around 7% of motorcycle-related injuries in our city occurred at homes in the second period. Al-Ain City is very unique. It spreads horizontally over a very wide region of 30 × 20 kilometers despite the small number of its population. Houses of residential areas are not allowed to be higher than four floors and they have wide fenced areas around them. These are not public areas and considered legally as part of the homes. Motorcycles which are used for sport activity or food delivery enter within these fences and may cause injuries. Understandably, developing an injury prevention strategy for these specific injuries needs special educational and legal considerations.

### Limitations of the study

Our study has certain limitations. First, it is from a single hospital which limits its generalizability in all the UAE. Second, there was a gap in our registry from 2007 to 2014 due to a lack of research funding. Third, data on helmet use and clothing (including boots) on the incident and circumstance that led to the crash were missing in our trauma registry. We did not evaluate other important factors such as rider's behavior, riding experience, motorcycle safety technology (like Anti-Lock Brake systems), biomechanism of injury, and road characteristics to give us more insights into the cause of reduced mortality. Fourth, our study had a small sample size which may cause type II statistical error. Nevertheless, these patients represent the majority of those treated over seven years in a city of three quarter of a million population. Furthermore, this small sample enabled us to collect high-quality prospective accurate data with minimum missing data. Finally, our study did not include patients treated at the emergency department who were discharged home, those with minor injuries who did not seek medical advice, and those who died on the streets; which has the risk of selection bias.

## Conclusions

The incidence of motorcycle injuries in our city dropped by almost 40% over the last 15 years. There was a significant reduction in the mortality of hospitalized motorcycle-injured patients despite increased anatomical severity of the head injuries. This is attributed to improvements in the trauma care system, including injury prevention, and both prehospital and in-hospital trauma care.

## Data Availability

There are no additional data available to share with the readers. Data can be shared with the Editor of the Journal if requested.

## Abbreviations

AIS: Abbreviate Injury Scale
EMS: Emergency Medical Service
GCS: Glasgow Coma Scale
HICs: High-income countries
ICU: Intensive care unit
IQR: Interquartile range
ISS: Injury Severity Score
NISS: New Injury Severity Score
RR: Respiratory rate
SBP: Systolic blood pressure
UAE: United Arab Emirates
WHO: World Health Organization
